# Bayesian inference in nonlinear growth models and hypothesis testing of sexual dimorphism in Black Utrerana chickens

**DOI:** 10.1016/j.psj.2025.105947

**Published:** 2025-10-03

**Authors:** Rafaela de Carvalho Salvador, Adriele Aparecida Pereira, Tales Jesus Fernandes

**Affiliations:** a Department of Statistics, Federal University of Lavras (UFLA), Lavras, MG, Brazil; b Institute of Applied Social Sciences, Federal University of Alfenas (UNIFAL-MG), Varginha, MG, Brazil

**Keywords:** Bayes factor, Bayesian inference, Growth curve, Hamiltonian Monte Carlo, Nonlinear regression

## Abstract

Poultry growth is a dynamic process that can be influenced by several factors, such as sexual dimorphism, which in most studies is described without the use of formal statistical tests. Understanding animal growth is vital for proper management and breedin. Nonlinear models are often used to describe animal growth, as they allow biological interpretation of the parameters and capture the sigmoid nature of the growth curve. Despite the widespread use of nonlinear modeling in poultry growth, no studies have been found that apply Bayesian inference to the estimation and comparison of parameters in chickens. This approach incorporates prior knowledge on the topic and produces probability distributions for the parameters, providing a clearer view of uncertainty and more robust inference. Therefore, to fill these knowledge gaps, this study aims to select the most suitable nonlinear model to characterize the body weight growth of male and female Black Utrerana chickens among the Logistic, Gompertz, Brody and von Bertalanffy models using the Bayesian methodology, which has not previously been applied to growth modeling in this species, and to evaluate the existence of sexual dimorphism in this breed. The data are mean weights (g) measured over age in days of Black male and female Utrerana (Spanish breed) chickens raised in a free-range system. The nonlinear models were fitted using the “brms” package of the R software, which implements the Hamiltonian Monte Carlo algorithm with No-U-Turn (NUTS) sampler. The Gompertz and von Bertalanffy models performed best in describing bird growth, regardless of sex. For clarity and conciseness, only the von Bertalanffy results are presented. After model selection, a Bayesian hypothesis test was performed based on the Savage-Dickey method in order to investigate sexual dimorphism in the growth parameters. The results indicated sexual dimorphism only in the asymptotic weight, being significantly higher in males. In conclusion, the Bayesian approach proved effective for modeling poultry growth, and our findings highlight that sexual dimorphism in Black Utrerana chickens is limited to asymptotic weight.

## Introduction

The poultry industry makes a significant contribution to the global economy, providing a vital source of protein worldwide through meat and egg production (Menchetti et al., 2024). According to the Food and Agriculture Organization of the United Nations (FAO, 2020), poultry meat accounted for almost 40 % of global meat production. In addition, global egg production has increased by 150 % over the last three decades (FAO, 2025).

Livestock production in general and domestic chicken production in particular plays a vital socio-economic role for people living in low-income countries of Africa and Asia (Mohamadinejad et al., 2024; Mohammadabadi et al., 2025). Domestic chickens are the most widely kept livestock species worldwide, largely due to their short generation interval and adaptability to diverse agro-ecological conditions (Mohammadifar and Mohammadabadi, 2017; Khabiri et al., 2025).

They provide not only high-quality protein but also a direct source of income for rural households, being particularly important for food security and poverty alleviation (Mohammadabadi et al., 2010; Mohammadifar and Mohammadabadi, 2018; Mohamadinejad et al., 2024; Mohammadabadi et al., 2024). This broad distribution and resilience are associated with valuable traits such as disease resistance, ability to withstand harsh environments, and efficient utilization of low-quality feeds (Shahdadnejad et al., 2016; Khabiri et al., 2023).

In this context, free-range poultry farming has gained prominence for combining food production with sustainability and animal welfare, constituting aspects which are increasingly valued by urban consumers (Bist et al., 2024; Ximenes, 2020).

An indigenous poultry breed adapted to the free-range system is Utrerana, originally from Andalusia (southern Spain), belonging to the Mediterranean light population. This breed, which is threatened with extinction, stands out for its hardiness, low prevalence of diseases and dual-purpose meat and egg production (González Ariza et al., 2019, 2021).

Poultry growth can be influenced by several factors, including sexual dimorphism, which is characterized by significant differences in body weight, growth patterns and gene expression between males and females (Tian et al., 2024). However, according to Neto et al. (2020), information on the growth and nutritional requirements of local breeds raised in free-range systems, such as Utrerana, is still scarce. This information is essential for sustainably preserving animals at risk of extinction.

Weight gain over time can be well adjusted by nonlinear models. The most widely used to describe poultry growth are the Logistic, Gompertz, von Bertalanffy and Brody models (Finco et al., 2016), which are widely applied to model the growth of free-range chickens (Iqbal et al., 2019; Neto et al., 2020; Mata-Estrada et al., 2020; González Ariza et al., 2021; Galán et al., 2023; Mancinelli et al., 2023)

Bayesian inference has been widely used to model poultry growth, especially quail (Firat et al., 2015; Finco et al., 2016; Rossi et al., 2017; Ozsoy et al., 2019; Faccin and Rossi et al., 2024). However, no records on the use of this methodology in studies with chickens were found. In addition to sample data (likelihood function), the Bayesian approach stands out for incorporating prior knowledge about the parameters (a *priori* distributions); the combination of this information results in access to the a *posteriori* distribution, which represents all probabilistic information about the parameters.

Furthermore, although visual or descriptive comparison between male and female growth curves, which have been used in most studies studying parameter comparisons (Salvador et al., 2025), may suggest the existence of differences, this simple comparison is not sufficient to infer the statistical significance of the observed variations. In this sense, Bayesian hypothesis tests become a relevant tool, as they enable comparing parameters in nonlinear models more directly and intuitively without the requirement of assumptions such as normality, which is common in classical tests (Wang and Liu, 2016; Salles et al., 2020). This is particularly important in assessing sexual dimorphism, allowing a more robust investigation of the differences in growth between males and females.

Thus, the objective of this work is to select the most appropriate nonlinear model to characterize the body weight growth of male and female Black Utrerana chickens among the Logistic, Gompertz, Brody and von Bertalanffy models using the Bayesian methodology, which has not previously been applied to growth modeling in this species, and to evaluate the existence of sexual dimorphism in this breed.

## Method

### The data set

The data were taken from the study by González Ariza et al. (2021). The weight-age information for this study was obtained from 698 chickens, composed of 421 females and 277 males of the Black Utrerana breed, raised in free-range conditions during the years 2018 and 2019 in a public hatchery located in the Provincial Agricultural Center of the Diputación de Córdoba, Spain.

A total of 36 average weight samples were collected, with 18 samples referring to the weight of males and 18 to the weight of females at 0, 7, 14, 21, 28, 42, 56, 70, 84, 112, 140, 168, 196, 224, 252, 280, 308, and 336 days after birth.

### Nonlinear model candidates

The non-linear models presented in Table 1 were considered for data analysis, which are the most suitable for modeling bird growth, according to Narinç et al. (2017).

**Table 1:** Nonlinear $Y_{i}$ equations (sigmoidal growth curves) investigated.

Function name	Mathematical expression
Logistic	$Y_{i}=\frac{A}{1+e^{\left[K*\left(B-t_{i}\right)\right]}}+\;\varepsilon_{i}$
Gompertz	$Y_{i}=A*e^{-e^{\left[K*\left(B-t_{i}\right)\right]}}+\varepsilon_{i}$
Von Bertalanffy	$Y_{i}=\;A*{\left[1-\;\frac{e^{\left[K*\left(B-t_{i}\right)\right]}}{3}\right]}^{3}+\varepsilon_{i}$
Brody	$Y_{i}=A*\left(1-Be^{-Kx_{i}}\right)+\varepsilon_{i}$

In which:$\;Y_{i}$ represents the bird’s body weight in grams (g); t is the age of the animal given in days (d); the A parameter can be interpreted as the asymptotic weight; B can be interpreted the abscissa of the inflection point (IP), often associated with the bird’s time of maturity, except in the Brody model in which B has no practical interpretation; K is a parameter related to the animal’s relative growth rate; it is assumed that the errors $\varepsilon_{i}$ are independent and identically distributed according to a normal distribution $\varepsilon_{i}∼N\left(0,\sigma^{2}\right).$

The parameters were estimated by Bayesian inference based on obtaining the *a posteriori* distribution - p(θ/y), which combines prior information (*a priori* distribution) - p(θ), with the data evidence (likelihood) - L(y/θ). This distribution is proportional to the product between the prior and the likelihood, according to Bayes’ Theorem, considering θ=(A,B,K):

For each model considered, the likelihood, L(y/θ), was assumed to be a normal distribution for the response variable $y_{i}$, with the mean given by the growth function and constant variance, meaning $y_{i}∼N\left(\mu_{i},\sigma^{2}\right)$ with $\mu_{i}$ defined by the model equation.

It is necessary to obtain the marginal *a posteriori* distribution to perform inference on a specific parameter, which requires integrating the joint distribution, p(θ/y), in relation to the other parameters. Since these integrals do not have an analytical solution in the considered models, the Hamiltonian Monte Carlo (HMC) algorithm was used to sample *a posteriori* and estimate the parameters of interest.

### Elicitation of a priori distributions

As previously mentioned, no records were found in the literature of articles that model chicken growth through Bayesian inference. However, studies with other birds, such as quail, provide an overview of the *a priori* distributions used in nonlinear growth models.

Ton et al. (2021) applied Gompertz, Logistic, Richards and Von Bertalanffy models using the inverse Gamma *a priori* distribution for the parameters, focusing on the K parameter which represents maturation. Rossi et al. (2017) adopted non-informative *a priori* distributions, with Normal distribution for the parameters related to the asymptotic weight in the Brody, Gompertz, Logistic and Von Bertalanffy models.

Finco et al. (2016) also used non-informative *a priori* distributions, with Normal distribution for the parameters in the Gompertz, Brody, Logistic and Von Bertalanffy models. When modeling growth with the Skew t-distribution, Faccin and Rossi (2024) used a *priori* t-distributions for the parameters. Ozsoy (2019) used Normal and Gamma distributions for the parameters of a hierarchical linear growth model in a study with quails. Normal distribution was adopted for A and B in this study due to the parametric space of the parameters, whose possible values adequately cover the expected range. In turn, the Gamma distribution was chosen for K, which ensures values consistent with its interpretation.

The hyperparameters for the a *priori* distributions were determined through a meta-analysis, which consisted of reviewing articles that described the weight gain of chickens of breeds with similar characteristics to Utrerana as a function of time through non-linear models, including the following: Villalba et al. (2007); Miguel et al. (2007); González-Ariza et al. (2019); Mata-Estrada et al. (2020) and Galán et al. (2023). This meta-analysis resulted in the values presented in Table 2.

**Table 2:** *Prior* distributions used for parameters A, B and K of nonlinear models.

Bayesian Parameters	*Prior* Distribution	Hyperparameter Values for Females	Hyperparameter Values for Males
A	Normal (μ,σ²)	μ = 1950; σ= 166.81	μ = 2800; σ = 290.62
B	Normal (μ,σ²)	μ = 62; σ = 5.32	μ=70;σ = 4.43
K	Gamma (α,β)	α = 25.60; β = 1496.80	α= 17,740; β = 1144.55

### Computational resources

The nonlinear models were fitted using the “*brms*” package designed to fit Bayesian regression models using the Stan probabilistic language with an R interface, which implements the Hamiltonian Monte Carlo (HMC) algorithm with a No-U-Turn (NUTS) sampler.

According to Bürkner (2018), “*brms*” acts as an interface between R and Stan, using similar formula syntax to “*lme4*”, facilitating the specification, fitting and interpretation of Bayesian models.

The Markov chains were run with 4,000 iterations each, totaling 4 chains, for parameter estimation. It is important to note that the 4 chains mentioned do not refer to the number of model parameters, but rather to the fact that HMC repeats the sampling process in 4 independent chains to verify convergence. The first 1,000 (25 %) samples of each simulation chain were discarded as a burn-in period.

Next, the convergence diagnosis was performed after all iterations on the 4 chains estimated by the HMC method. It was then verified through graphical analysis of the traceplot graph for the 4 Markov chains and the Gelman-Rubin potential scale reduction factor ($\hat{R}$).

After adjusting the 4 nonlinear models to the data of males and females, a comparison was made between them using the following indices: estimated Expected log Pointwise Predictive Density (ELPD); value of the Leave-one-out Cross-Validation Information Criterion (LOO-IC); and the Watanabe-Akaike Information Criterion (WAIC). A detailed description can be found in Vehtari, Gelman and Gabry (2017).

Lower values of the LOO-IC and WAIC indices indicate the model that best fits the data; in addition, higher ELPD values show the best predictive capacity. In turn, the same model was selected for each sex using these indices.

The last step consisted of evaluating sexual dimorphism through a Bayesian hypothesis test using the Savage – Dickey method. This involved testing the equality of each parameter between the sexes by comparing the estimated values for females and males in the model previously selected as the most appropriate to describe growth.

The hypotheses tested for parameter A(asymptotic weight), B (abscissa of the inflection point) and K (parameter related to growth speed) were respectively:

### Bayesian hypothesis testing

Bayesian hypothesis testing is formulated based on the *a posteriori* probability ratio of the hypotheses $H_{0}$ (null hypothesis) and $H_{1}$ (alternative hypothesis), given the data evidence x. The Bayes ratio between the hypotheses is given by:

### Savage - Dickey method

The Savage - Dickey method is a specific approach to calculating the Bayes factor used to compare two hypotheses: the null hypothesis ($H_{0}$) and the alternative hypothesis ($H_{1}$). For a parameter θ, if $H_{0}$ assumes that $\theta =\theta_{0}$ and $H_{1}$ assumes that $\theta \neq \theta_{0}$.

The Bayes factor ($BF_{01})$ calculated using the Savage - Dickey density is the ratio of the *a posteriori* density for $\theta_{0}\;$at the value $\theta_{0}$, under the alternative hypothesis $H_{1}$ and the prior density for θ at the same value, defined as: $p(\theta =\theta_{0}|x,H_{1})$ is the *a posteriori* density at the specific value $\theta_{0}$ under the alternative hypothesis $H_{1}$ after observing the data x. And $p(\theta \neq \theta_{0}|H_{1})$ is the *a priori* density at the specific value $\theta_{0}$ under the alternative hypothesis $H_{1}$; for more details see Wagenmakers et al. (2010). The interpretation of the Bayes factors was done according to the guidelines proposed by Jeffreys (1961).

## Results and discussion

### MCMC chain convergence diagnosis

Potential scaling factors (i.e. Gelman-Rubin $\hat{R}\;$statistics) assess the convergence of Markov Chain Monte Carlo (MCMC) models in Bayesian models. They indicate whether the variation between chains and the variation within chains suggest that convergence has been achieved.

Based on the values obtained for the potential scaling factor ($\hat{R}$) presented in Table 3, there was no evidence of non-convergence in the chains sampled by the MCMC (HMC) method. The $\hat{R}\;$estimates remained close to 1 - within the recommended range (0.99 to 1.01) - which is indicative of good convergence of the Markov chains in all of the considered models, according to the criterion proposed by Gelman and Rubin (1992).

**Table 3:** Estimation of $\hat{R}$ (potential scale reduction factor) for the parameters of the nonlinear Gompertz, Logistic, von Bertalanffy and Brody models adjusted to the weight of Utrerana Black variety chickens, males and females, over time.

		$\hat{R}$		
	Gompertz	Logistic	von Bertalanffy	Brody
$A_{\mathrm{female}}$	1.0005265	1.000341	1.0002257	1.000791
$B_{\mathrm{female}}$	0.9999145	1.001159	1.0003354	1.000328
$K_{\mathrm{female}}$	1.0000905	1.000462	0.9999711	1.000446
$A_{\mathrm{male}}$	1.000186	0.9999932	1.000113	1.000305
$B_{\mathrm{male}}$	1.000481	1.0005122	1.000382	1.000368
$K_{\mathrm{male}}$	1.000025	1.0011714	1.000181	1.000136

This convergence suggests that the parameter samples are reliable and representative of the *a posteriori* distribution, which strengthens the validity of the parameter estimates and the models’ fit to the chicken growth data. Furthermore, the graphical analysis of the traceplot (a graphical tool which displays the value of the parameters of interest throughout the chain iterations) was stationary for all tested models and for both sexes, which is a strong indication of white noise, as described by Robert and Casella (2004).

### Model comparison and selection

Table 4 shows the results obtained for the indexes considered, including the Expected log Pointwise Predictive Density (ELPD), Leave-one-out Cross-Validation Information Criterion (LOO-IC) and Watanabe-Akaike Information Criterion (WAIC), considering the nonlinear Bayesian models fitted to the growth data of male and female Black Utrerana chickens.

**Table 4:** Comparative indices for nonlinear models adjusted to growth data of Utrerana breed chickens, female and male of the Black variety (values in parentheses represent the standard error (SD) of the estimates).

Models	LOO-IC ¹ (SE)	WAIC ² (SE)	$ELPD_{LOO}$³ (SE)
		**FEMALE**	
**Gompertz**	203.6 (12.7)	201.7 (11.0)	−101.8 (6.4)
**Logistics**	241.0 (4.8)	240.3 (4.2)	−120.5 (2.4)
**von Bertalanffy**	203.1 (6.2)	202.5 (5.9)	**−**101.5 (3.1)
**Brody**	313.7 (3.6)	313.6 (3.6)	−156.9 (1.8)
		**MALE**	
**Gompertz**	202.9 (5.8)	202.9 (5.6)	−101.5 (2.9)
**Logistics**	233.9 (3.0)	233.7 (2.9)	−116.9 (1.5)
**von Bertalanffy**	205.5 (6.6)	204.9 (6.3)	−102.7 (3.3)
**Brody**	319.6 (3.9)	319.6 (3.9)	−159.8 (1.9)

The Gompertz model presented the lowest LOO-IC, WAIC and $ELPD_{LOO}$ values for males, while the von Bertalanffy model had a slightly better performance for females. Thus, it is observed that each model stood out for one of the sexes, although the differences between the comparative indexes were small.

More precisely, the differences between the Gompertz and von Bertalanffy models were much smaller than the associated uncertainty (less than one standard error), indicating that both models describe the data equally well.

Studies such as those by Menchetti et al. (2024); Galán et al. (2023), González Ariza et al. (2019) and Rizzi et al. (2013) reinforce the good fit of the Gompertz model to chicken growth data. On the other hand, studies such as those by González-Ariza et al. (2021); Finco et al. (2016) and Darmani Kuhi et al. (2002) recognize the von Bertalanffy model as a robust alternative, with good performance to represent weight gain over time.

Also according to Darmani Kuhi et al. (2002), the Gompertz equation presents the possible limitation of a fixed inflection point, occurring at 1/e (= 0.368) times the asymptotic weight, while the von Bertalanffy model has a flexible (variable) IP that occurs between 0.296 and 0.368 times the asymptotic weight.

Therefore, considering that the application of the Savage–Dickey method requires the use of the same model for the groups being compared, and given that both Gompertz and von Bertalanffy provide an equally good description of the data and lead to the same biological conclusions, we chose to present only the von Bertalanffy results in the Bayesian comparisons of growth parameters, for clarity and conciseness.

### Parameter estimates and assessment of sexual dimorphism

Table 5 presents the mean estimates of the von Bertalanffy model parameters fitted to the growth data of male and female Black Utrerana chickens, with respective 95 % HPD credibility intervals and standard deviations.

**Table 5:** Average interval estimates of the nonlinear Bayesian von Bertalanffy growth model fitted to the growth data of female and male Utrerana chickens, changes in 95 % Highest Posterior Density (HPD) and standard deviation (SD).

Parameter	Estimate	$HPD_{Lower}$(95 %)	$HPD_{Upper}\left(95\%\right)$	SD
		**FEMALE**		
**A**	2109.8286	2033.9003	2184.5512	38.1790
**B**	64.2118	60.0749	68.2307	2.0218
**K**	0.0152	0.0135	0.0173	0.0010
		**MALE**		
**A**	2560.5027	2481.7118	2648.7857	41.9023
**B**	65.1733	61.8542	68.8085	1.7125
**K**	0.0156	0.0138	0.0174	0.0009

The values obtained for parameter A (asymptotic weight) were approximately 2,110 g for females and 2,560 g for males. These results are in agreement with the literature data: Cabello et al. (2009) reported an average weight of 2,504 g for the Black variety of the breed, regardless of sex; while González Ariza et al. (2021) indicated specific maturity weights of 2,033 g for hens and 2,606 g for roosters. Then the estimates for parameter B, which represents the IP abscissa of the growth curve (age of greatest weight gain), were close to 64 days for both sexes, which is a value consistent with that observed by Plato-Casado et al. (2023) who identified this point at 59 days in the Black variety. In turn, parameter K, related to growth speed, presented values around 0.015 for males and females, close to the 0.014 reported by González Ariza et al. (2021) for the same breed.

Table 5 shows that the credibility intervals for parameter A do not overlap between sexes, suggesting a significant difference in this parameter. In contrast, the HPD intervals for parameters B and K overlap, indicating no clear evidence of a difference between males and females.

**Fig. 1:**
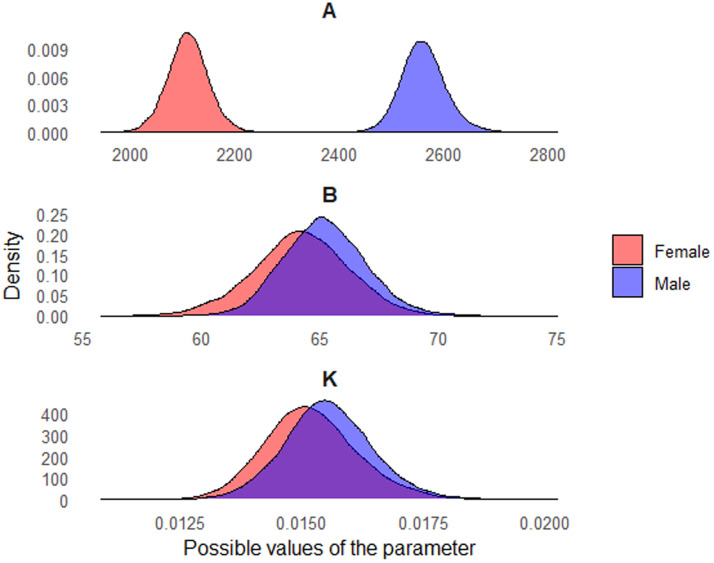
Posterior distributions of the von Bertalanffy model parameters estimated for male and female Utrerana chickens.

Fig. 1 reinforces these findings, illustrating the marginal a *posteriori* distributions of parameters A, B, and K by sex. These distributions enable visualizing the uncertainty associated with each estimate and highlight the separation in parameter A, in addition to the proximity between the curves in parameters B and K. This demonstrates the advantage of the Bayesian approach, which in addition to providing point estimates, also enables a more complete and interpretable analysis of the variability in the parameters.

This level of detail is especially useful for managing underexplored breeds, such as Utrerana, as it enables understanding the growth pattern of the animals with a smaller margin of error. According to Gelman et al. (2014), the a *posteriori* distributions provide a rich description of the phenomenon studied, allowing for more precise and reliable estimates.

In addition, as highlighted by Salles et al. (2020), these distributions allow direct comparisons between parameters of different groups, such as sex, which was essential for assessing sexual dimorphism in this study.

**Fig. 2:**
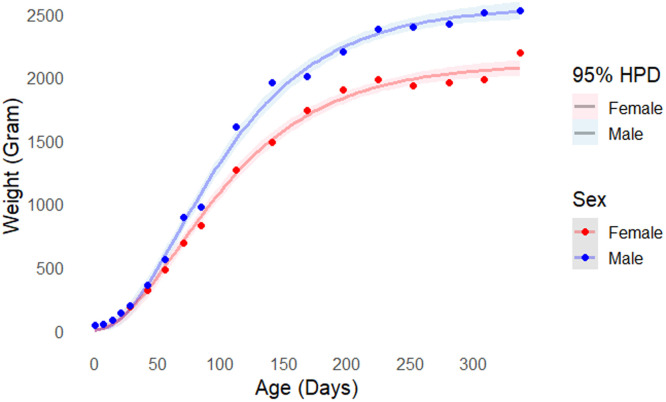
Growth curves adjusted with 95 % credible intervals (shaded areas). Dots represent observed data for males (blue) and females (red).

Complementing this information, Fig. 2 presents the fitted growth curves for the male and female black Utrerana chickens, with their respective credibility regions (HPD 95 %). The fits were made using the a *posteriori* mean of the parameters.

Although the HPD intervals and a *posteriori* distributions provide a detailed description of the uncertainty in the estimates, they are not sufficient to assess the plausibility of a hypothesis. This limitation is overcome by the Bayesian hypothesis test, which uses the Bayes factor to quantify the strength of evidence between hypotheses and even allows the null hypothesis to be accepted based on the data (Wagenmakers et al., 2010).

Therefore, statistical tests, such as the Bayesian hypothesis test, are necessary to statistically confirm the existence of sexual dimorphism in certain parameters. The simple overlap or visual separation in the curves may be indicative, but does not constitute statistical evidence in itself.

Table 6 presents the estimates of the Bayesian hypothesis tests applied to the von Bertalanffy model parameters fitted to the growth of male and female Black Utrerana chickens. The null hypothesis ($H_{0}$) evaluated in each case assumes equality between the sexes for a given parameter. Unlike the frequentist approach, Bayesian inference allows us to calculate the *a posteriori* probability of $H_{0}$ being true, providing more direct and interpretable conclusions (Rochon et al., 2012).

**Table 6:** Estimates from Bayesian hypothesis testing.

Hypothesis $H_{0}$	$BF_{01}$	$P(H_{0}|x)$
$A_{male}-A_{female}=0$	0.0009985655	0.0009975694
$B_{male}-B_{female}=0$	4.870551	0.8296582
$K_{\mathrm{male}}-K_{\mathrm{fema}\mathrm{le}}=0$	3.848496	0.7937505

Table 6 shows that the *a posteriori* probability of there being no difference between the sexes in terms of asymptotic weight is approximately 0.1 %. This value represents extremely strong evidence against the null hypothesis ($H_{0}$), unequivocally indicating that males and females have different asymptotic weights. Considering that males reach greater weight in adulthood, this result suggests that they may be more suitable for meat-oriented production systems, while females, with lower asymptotic weights but similar maturity, may be more focused on egg production.

Several studies have shown sexual dimorphism in poultry growth, as demonstrated by Aggrey (2002), Miguel et al. (2007) and Galán et al. (2023) as well as in specific studies with the Utrerana breed (González Ariza et al., 2019 and González Ariza et al., 2021). These studies fit growth curves separately for males and females and discussed the differences based on point estimates of the parameters. However, this simple comparison is not sufficient to infer statistical significance from the observed differences.

Applying statistical tests is essential to verify whether the observed differences are real or attributed to chance, as illustrated by Cajal and Francesch (2014), who used ANOVA via GLM, and Nahashon et al. (2006), who compared point estimates of the parameters of sex-fitted growth models, both without finding significant differences between males and females in terms of growth in chickens.

Next, parameter B (associated with the IP abscissa of the growth curve), the Bayes factor $BF_{01\;}$= 4.87 indicates substantial evidence in favor of $H_{0}$, with an *a posteriori* probability of 83 %. According to Jeffreys’ interpretive scale, here expressed in terms of $BF_{01}$ (Jeffreys, 1961; Jarosz and Wiley, 2014; see Table 7), values between 3 and 10 are typically classified as providing *substantial evidence*, which contextualizes the present result. This suggests that the data support the absence of a difference between the sexes regarding this parameter (Table 6).

**Table 7:** Interpretation of Bayes factor values according to Jeffreys’ scale, expressed here in terms of BF₀₁ (adapted from Jeffreys, 1961; Jarosz and Wiley, 2014).

Bayes Factor $\left(BF_{01}\right)$	Interpretation (evidence in favor of $H_{0}$)
1−3	Anecdotal / Weak evidence
3−10	Substantial evidence
10−30	Strong evidence
30−100	Very strong evidence
>100	Decisive evidence

Note: Jeffreys’ interpretive scale is traditionally expressed in terms of $BF_{10}$ (evidence for $H_{1}$ over $H_{0}$). For clarity, the thresholds are presented here in terms of $BF_{01}$, the reciprocal measure ($BF_{01}$= 1/$BF_{10}$).

Although Plata-Casado et al. (2023) reported that Utrerana females reach IP earlier, and the point estimates of this study indicate a similar trend, the absence of formal statistical tests in the literature and the overlap of credibility intervals between the sexes indicate that there is no robust evidence to confirm this difference. The scarcity of studies on the breed’s growth curve reinforces the need for more rigorous analyses based on statistical inference.

Then the parameter K (which describes the growth rate), the Bayes factor $BF_{01}$ = 3.85 and the a *posteriori* probability of 79 % also indicate evidence in favor of the null hypothesis. According to Jeffreys’ interpretive scale, here expressed in terms of $BF_{01}$ (Jeffreys, 1961; Jarosz and Wiley, 2014; see Table 7), this value falls within the range typically classified as *substantial evidence* for $H_{0}$.

Since the hypotheses tested refer to equality between the sexes (Table 6), the use of Bayesian inference proved to be especially appropriate. Unlike the frequentist approach, which focuses on rejecting the null hypothesis ($H_{0}$), Bayesian inference allows us to directly quantify the plausibility of hypotheses, providing a measure of support for $H_{0}$ and $H_{1}$ (Rouder et al., 2009; Wagenmakers et al., 2010). In this sense, while the non-rejection of $H_{0}\;$in the classical context only indicates the absence of evidence to reject it, the Bayesian approach allows a more direct assessment of the probability of equality between the parameters.

A limitation of our study is that the analysis was performed using mean weights rather than individual-level data, which may reduce variance information. However, because Bayesian inference treats parameters as random variables with probability distributions that explicitly account for estimation uncertainty, this issue is mitigated compared with classical approaches.

## Conclusion

Bayesian inference provided a clearer understanding of the uncertainties and differences in growth parameters through posterior distributions and formal tests. The von Bertalanffy and Gompertz models were the most appropriate for characterize the body weight growth of Black Utrerana chickens. Sexual dimorphism was observed only in asymptotic weight, which was higher in males, with no significant differences in the other parameters between sexes. These results contribute to the conservation of the endangered Utrerana breed by informing reproductive planning. Males are strategic for niche meat markets, whereas females can be maintained for dual-purpose use.

## Declaration of AI and AI-assisted technologies in the writing process

During the preparation of this work, the authors used ChatGPT (developed by OpenAI) to improve the clarity of the writing and to solve technical issues related to the data analysis scripts. After using this tool, the authors reviewed and edited the content as necessary and assume full responsibility for the content of the publication.

## CRediT authorship contribution statement

**Rafaela de Carvalho Salvador:** Writing – original draft, Visualization, Validation, Software, Methodology, Investigation, Formal analysis, Data curation, Conceptualization. **Adriele Aparecida Pereira:** Writing – review & editing, Supervision, Software, Conceptualization. **Tales Jesus Fernandes:** Writing – review & editing, Supervision, Methodology, Conceptualization.

## Disclosures

The authors declare that they have no known competing financial interests or personal relationships that could have appeared to influence the work reported in this paper.

## References

[bib68] Aggrey S.E. (2002). Comparison of three nonlinear and spline regression models for describing chicken growth curves. Poult. Sci..

[bib0005] Bürkner P. (2018). Advanced Bayesian multilevel modeling with the R package brms. R. J..

[bib0003] Bist R.B., Bist K.., Poudel S., Subedi D., Yang X., Paneru B., Mani S., Wang D., Chai L. (2024). Sustainable poultry farming practices: a critical review of current strategies and future prospects. Poult. Sci..

[bib0006] Cabello A., León J.M., Melo P., Doctor J. (2009). Estudio de la curva de crecimiento en la gallina Utrerana. FEAGAS.

[bib0007] Cajal J.R., Francesch A. (2014). Caracterización productiva de la gallina de Sobrarbe Arch. Zootec.

[bib0008] Darmani Kuhi H., Kebreab E., Lopez S., France J. (2002). A derivation and evaluation of the von Bertalanffy equation for describing growth in broilers over time. J. Anim. Feed Sci..

[bib0010] Faccin M.Z., Rossi R.M. (2024). Bayesian modeling of the Gompertz curve for meat quails growth data considering different error distributions. Braz. J. Biom..

[bib0009] FAO (2025). https://www.fao.org/poultry-production-products/production/en/.

[bib60] FAO, 2020. Gateway to Poultry Production and Products – Production. Food and Agriculture Organization of the United Nations. Accessed Apr. 2025. https://www.fao.org/poultry-production-products/production/en/?utm_source.

[bib0013] Finco E.M., Marcato S..M., Furlan A.C., Rossi R.M., Grieser D.O., Zancanela V., Oliveira T.M.M., Stanquevis C.E. (2016). Adjustment of four growth models through bayesian inference on weight and body nutrient depositions in laying quail. Rev. Bras. Zootec..

[bib62] Firat M.Z., Karaman E., Başar E.K., Narinc D. (2015). Bayesian analysis for the comparison of nonlinear regression model parameters: an application to the growth of Japanese quail. Braz. J. Poult. Sci..

[bib0016] Galán I., Arando A., González A., Navas F.J., Salgado J.I., Díaz E., Peláez M.P., León J.M., Delgado J.V., Camacho M.E. (2023). Caracterização de las curvas de crecimiento biológico de la gallina Andaluza Azul, una raza local amenazada. Arch. Zootec..

[bib65] Gelman A., Rubin D.B. (1992). Inference from iterative simulation using multiple sequences. Statist. Sci.

[bib67] Gelman A., Carlin J.B., Stern H.S., Dunson D.B., Vehtari A., Rubin D.B. (2014). Bayesian Data Analysis.

[bib0019] González Ariza A., Nogales S., Navas-González¸ F.J., Delgado J.V., León J.M., Barba C.J., Arando A., Camacho M.E. (2019). Estudio preliminar de caracterización del crecimiento de la raza aviar gallina Utrerana. Actas Iberoam. Conserv. Anim..

[bib0018] González Ariza A., Baena S.N., Lupi T.M., Arbulu A.A., Navas-González F.J., León Jurado J.M., Camacho Vallejo M.E. (2021). Characterisation of biological growth curves of different varieties of an endangered native hen breed kept under free range conditions. Ital. J. Anim. Sci..

[bib0020] Iqbal F., Eyduran E., Mikail N., Sarıyel V., Huma Z.E., Aygün A., Keskin İ. (2019). A Bayesian approach for describing the growth of Chukar partridges. Eur. Poult. Sci..

[bib0022] Jarosz A.F., Wiley J. (2014). What are the odds? A practical guide to computing and reporting Bayes factors. J. Probl. Solving.

[bib0023] Jeffreys H. (1961).

[bib0025] Khabiri A., Toroghi R., Mohammadabadi M.R., Tabatabaeizadeh S.E. (2023). Introduction of a Newcastle disease virus challenge strain (sub-genotype VII. 1.1) isolated in Iran. Vet. Res. Forum.

[bib0024] Khabiri A., Toroghi R., Mohammadabadi M.R., Tabatabaeizadeh S.E. (2025). Whole genome sequencing and phylogenetic relative of a pure virulent Newcastle disease virus isolated from an outbreak in northeast Iran. Lett. Appl. Microbiol..

[bib0029] Mancinelli A.C., Menchetti L.., Birolo M., Bittante G., Chiattelli D., Castellini C. (2023). Crossbreeding to improve local chicken breeds: predicting growth performance of the crosses using the Gompertz model and estimated heterosis. Poult. Sci..

[bib0027] Mata-Estrada A., González-Cerón F., Pro-Martínez A., Torres-Hernández G., Bautista Ortega J., Becerril-Pérez C.M., Vargas-Galicia A.J., Sosa-Montes E. (2020). Comparison of four nonlinear growth models in Creole chickens of Mexico. Poult. Sci..

[bib0030] Menchetti L., Birolo M., Mugnai C., Mancinelli A.C., Xiccato G., Trocino A., Castellini C. (2024). Effect of genotype and nutritional and environmental challenges on growth curve dynamics of broiler chickens. Poult. Sci..

[bib0031] Miguel J.A., Asenjo B.., Ciria J., Calvo J.L. (2007). Growth and lay modelling in a population of Castellana Negra native Spanish hens. Br. Poult. Sci..

[bib0034] Mohamadinejad F., Mohammadabadi M.R., Roudbari Z., Siahkouhi S.E., Babenko O., Klopenko N., Borshch O., Starostenko I., Kalashnyk O., Soumeh E.A. (2024). Analysis of liver transcriptome data to identify the genes affecting lipid metabolism during the embryonic and hatching periods in ROSS breeder broilers. J. Livest. Sci. Technol..

[bib0037] Mohammadabadi M.R., Nikbakhti M.., Mirzaee H.R., Shandi A., Saghi D.A., Romanov M.N., Moiseyeva I.G. (2010). Genetic variability in three native Iranian chicken populations of the Khorasan province based on microsatellite markers. Russ. J. Genet..

[bib0035] Mohammadabadi M.R., Akhtarpoor A.., Khezri A., Babenko O., Stavetska R.V., Tytarenko I., Ievstafiieva Y., Buchkovska V., Slynko V., Afanasenko V. (2024). The role and diverse applications of machine learning in genetics, breeding, and biotechnology of livestock and poultry. Agric. Biotechnol. J..

[bib0036] Mohammadabadi M.R., Afsharmanesh M.., Khezri A., Kheyrodin H., Babenko Ivanivna O., Borshch O., Kalashnyk O., Nechyporenko O., Afanasenko V., Slynko V., Usenko S. (2025). Effect of mealworm on GBP4L gene expression in the spleen tissue of Ross Broiler chickens. Agric. Biotechnol. J..

[bib0032] Mohammadifar A., Mohammadabadi M.R. (2017). The effect of uncoupling protein polymorphisms on growth, breeding value of growth and reproductive traits in the fars indigenous chicken. Iran. J. Appl. Anim. Sci..

[bib0033] Mohammadifar A., Mohammadabadi M.R. (2018). Melanocortin-3 receptor (MC3R) gene association with growth and egg production traits in Fars indigenous chicken. Malays. Appl. Biol..

[bib0040] Nahashon S.N., Aggrey S.E., Adefope N.A., Amenyenu A. (2006). Modeling growth characteristics of meat-type Guinea fowl. Poult. Sci..

[bib0041] Narinç D., Öksüz Narinç N., Aygün A. (2017). Growth curve analyses in poultry science. World's Poult. Sci. J..

[bib61] Neto V.I., Barbosa F.J.V., Campelo J.E.G., Sarmento J.L.R. (2020). Non-linear mixed models in the study of growth of naturalized chickens. R. Bras. Zootec..

[bib0042] Ozsoy A.N. (2019). Genetic parameter estimations of bayesian hierarchical linear and nonlinear growth curves in Japanese quails. Fresenius Environ. Bull..

[bib0043] Plata-Casado A., García-Romero C., González-Redondo P.O. (2023). Selection and current status of the Utrerana chicken breed: a review. Animals.

[bib0044] Rizzi C., Contiero B., Cassandro M. (2013). Growth patterns of Italian local chicken populations. Poult. Sci..

[bib0045] Robert C.P., Casella G. (2004).

[bib0046] Rochon J., Gondan M., Kieser M. (2012). A Bayesian approach to hypothesis testing: why p-values are not enough. J. Stat. Plan. Inference.

[bib0047] Rossi R.M., Grieser D..O., Conselvan V.A., Marcato S.M. (2017). Growth curves in meat-type and laying quail: a Bayesian perspective. Semina.

[bib0048] Rouder J.N., Speckman P.L., Sun D., Morey R.D. (2009). Bayesian t tests for accepting and rejecting the null hypothesis. Psychon. Bull. Rev..

[bib0049] Salles T.T., Beijo L.A., Nogueira D.A., Almeida G.C., Martins T.B., Gomes V.S. (2020). Modeling the growth curve of Santa Inês sheep using a Bayesian approach. Livest. Sci..

[bib0050] Salvador R.C., Gonzaga N.A., Azarias E.C.P., Pereira A.A., Fernandes T.J. (2025). Test of equality of parameters in growth curves of black Castellana chickens. Semina.

[bib0051] Shahdadnejad N., Mohammadabadi M.R., Shamsadini M. (2016). Typing of Clostridium perfringens isolated from broiler chickens using multiplex PCR. Genet. Third Millenn..

[bib0052] Tian J., Tan L., Wei S., Zhu W., Ji C., Yao Z., Xu Y., Nie Q. (2024). Using multiomics to explore the weight differences between genders in Muscovy ducks. Poult. Sci..

[bib64] Ton A.P.S., Laureano M.M.M., Araújo S.I., Menezes F.L., Araújo C.V. (2021). Adjustment of growth curves in cutting quails by Bayesian inference. Res. Soc. Dev..

[bib0053] Vehtari A., Gelman A., Gabry J. (2017). Practical Bayesian model evaluation using leave-one-out cross-validation and WAIC. Stat. Comput..

[bib0055] Villalba D., Francesch A., Pons A., Bustamante J., Espadas M., Cubiló D. (2007). Resultados de puesta y crecimiento de una población de gallinas de raza menorca. Arch. Zootec..

[bib0056] Wagenmakers E.J., Lodewyckx T.., Kuriyal H., Grasman R. (2010). Bayesian hypothesis testing for psychologists: a tutorial on the Savage-Dickey method. Cogn. Psychol..

[bib63] Wang M., Liu G. (2016). A simple two-sample Bayesian *t*-Test for hypothesis Testing. Am. Stat..

[bib0057] Ximenês L.F. (2020).

